# Characterization of immortalized human brown and white pre-adipocyte cell models from a single donor

**DOI:** 10.1371/journal.pone.0185624

**Published:** 2017-09-28

**Authors:** Lasse K. Markussen, Marie S. Isidor, Peter Breining, Elise S. Andersen, Nanna E. Rasmussen, Louise I. Petersen, Steen B. Pedersen, Bjørn Richelsen, Jacob B. Hansen

**Affiliations:** 1 Department of Biology, University of Copenhagen, Copenhagen, Denmark; 2 Department of Clinical Medicine, Aarhus University, Aarhus, Denmark; 3 Department of Endocrinology and Internal Medicine, Aarhus University Hospital, Aarhus, Denmark; University of Barcelona, Faculty of Biology, SPAIN

## Abstract

Brown adipose tissue with its constituent brown adipocytes is a promising therapeutic target in metabolic disorders due to its ability to dissipate energy and improve systemic insulin sensitivity and glucose homeostasis. The molecular control of brown adipocyte differentiation and function has been extensively studied in mice, but relatively little is known about such regulatory mechanisms in humans, which in part is due to lack of human brown adipose tissue derived cell models. Here, we used retrovirus-mediated overexpression to stably integrate human telomerase reverse transcriptase (TERT) into stromal-vascular cell fractions from deep and superficial human neck adipose tissue biopsies from the same donor. The brown and white pre-adipocyte cell models (TERT-hBA and TERT-hWA, respectively) displayed a stable proliferation rate and differentiation until at least passage 20. Mature TERT-hBA adipocytes expressed higher levels of thermogenic marker genes and displayed a higher maximal respiratory capacity than mature TERT-hWA adipocytes. TERT-hBA adipocytes were UCP1-positive and responded to β-adrenergic stimulation by activating the PKA-MKK3/6-p38 MAPK signaling module and increasing thermogenic gene expression and oxygen consumption. Mature TERT-hWA adipocytes underwent efficient rosiglitazone-induced ‘browning’, as demonstrated by strongly increased expression of UCP1 and other brown adipocyte-enriched genes. In summary, the TERT-hBA and TERT-hWA cell models represent useful tools to obtain a better understanding of the molecular control of human brown and white adipocyte differentiation and function as well as of browning of human white adipocytes.

## Introduction

For decades it was believed that the existence of brown adipose tissue (BAT) in humans was limited to newborns or patients with catecholamine-secreting neuroendocrine tumors (e.g. pheochromocytomas, paragangliomas and neuroblastomas), and that BAT in normal children regressed to be absent in adulthood. However, metabolically active BAT depots have been detected in adult humans in the cervical, supraclavicular, interscapular and paravertebral areas by positron emission tomography/computed tomography using the deoxyglucose analog ^18^F-fluorodeoxyglucose [1–4]. The existence of brown-like (also called beige, brite or inducible brown) adipocytes in human BAT have been observed in several studies [5–9]. Brown-like adipocytes arise in response to physiological states such as cold exposure [10] and cancer cachexia [11], as well as in response to treatment with β-adrenergic agonists and thiazolidinedione agonists of peroxisome proliferator-activated receptor γ (PPARγ) [12–14].

BAT is a promising therapeutic target for combating obesity and related metabolic disorders due to its inherent capacity for dissipating energy as heat through the action of uncoupling protein 1 (UCP1) [15]. In the last 20 years, a large number of mouse models have documented the potential of BAT to combat metabolic dysfunction. The molecular circuitry of mouse brown adipocyte differentiation and function has been studied intensely due to easily accessible primary cell cultures and the existence of a number of useful cell models. The selection of human adipocyte cell models derived from adult BAT is limited, thereby hampering studies of the molecular control of human brown adipocyte differentiation and function.

Human and mouse BAT mitochondria have comparable UCP1 function [16], but intrinsic differences between human and mouse adipocytes have been observed in the response to e.g. glucocorticoids [17], retinoic acid [18], adrenocorticotropic hormone [19] and tumor necrosis factor [20], demonstrating that not all findings in mouse models can be extrapolated to the human situation and highlighting the relevance of proper human-derived cell models to study human physiology and pathophysiology.

A number of human white adipocyte cell models have been described, including unipotent models such as LiSa-2 [21], SGBS [22], Chub-S7 [23] and LS14 [24], but also multipotent models like hMADS [25]. The availability of primary human white adipocytes from liposuctions has made primary white adipocyte cultures an easily accessible supplement to cell models. Contrarily, primary human brown adipocytes are mainly available by surgical procedures where cancer is suspected in cervical and supraclavicular areas, and the amount of material is often limited. Only two studies have reported the generation of immortalized BAT cell models obtained from adult humans [26, 27]. Shinoda et al. established sub-cloned human brown-like and white pre-adipocyte cell models immortalized with simian virus 40 large T antigen from non-matched patients [26], while Xue et al. established patient-matched, sub-cloned human brown-like and white pre-adipocyte cell models immortalized with telomerase reverse transcriptase (TERT) [27]. The characterization of these derived cell clones elegantly demonstrated the heterogeneity of human BAT. The subcloning strategy also allowed the identification of adipogenic cell clones highly responsive to cAMP or bone morphogenetic protein 7 [26, 27]. Studies with immortalized clonal lines can provide very important information but are unlikely to represent the response of the intact tissue, e.g. due to the absence of heterogeneity in clonal cell models. Although speculative, cell cultures that have not undergone subcloning, i.e. are polyclonal, might elicit a response to a stimulus that is more closely reflecting the response of the intact tissue to the same stimulus.

Here we report the generation of TERT-immortalized polyclonal brown and white pre-adipocyte cell models from the same patient. The resulting cell models, TERT-hBA and TERT-hWA, exhibited high proliferative and adipogenic capacities up to at least passage 20. TERT-hBA adipocytes maintained classical features of thermogenic adipocytes, being UCP1-positive as well as displaying higher levels of mitochondrial markers and higher maximal respiratory capacity compared with TERT-hWA adipocytes. TERT-hBA adipocytes were highly receptive and responsive to β-adrenergic stimuli, as demonstrated by activation of the protein kinase A (PKA)-mitogen-activated protein kinase (MAPK) kinase 3/6 (MKK3/6)-p38 MAPK signaling module and increased thermogenic gene expression and oxygen consumption. Finally, mature TERT-hWA adipocytes underwent browning in response to treatment with rosiglitazone.

## Methods and materials

### Isolation and immortalization of human brown and white pre-adipocytes

**Fig 1:**
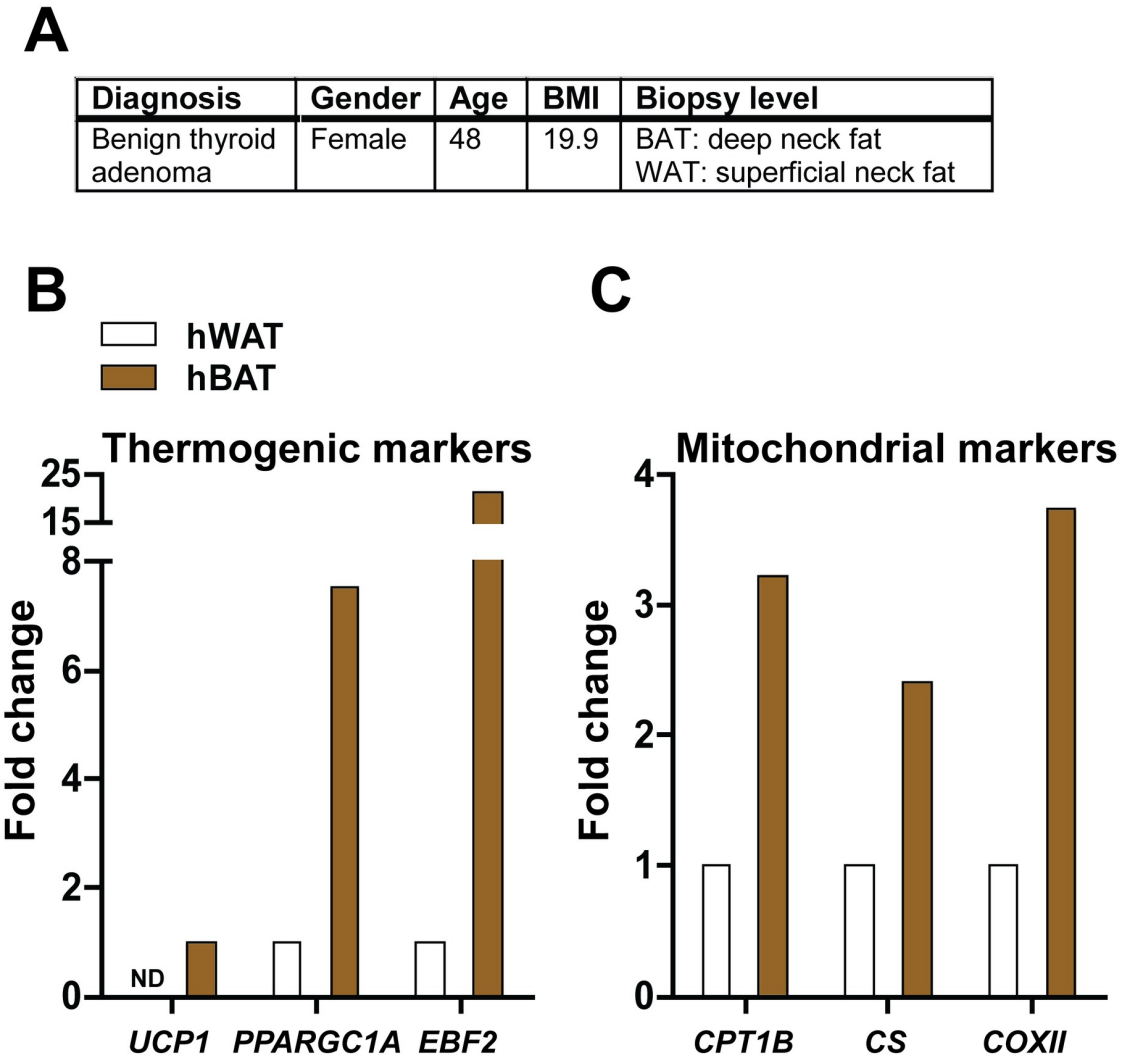
Subject and biopsy characterization. **(A)** Patient information. **(B)** Relative mRNA levels of the thermogenic genes *UCP1*, *PPARGC1A* and *EBF2* in hBAT and hWAT. **(C)** Relative mRNA levels of the mitochondrial genes *CPT1B*, *CS* and *COXII* in hBAT and hWAT. In (B) and (C), expression levels were normalized to *TBP* levels. The normalized expression in hWAT was set to 1, except for *UCP1* in which hBAT was set to 1. Data represent the mean of a technical duplicate without error bars, since only one patient was included. Statistical analyses were not applied.

Stromal vascular fractions (SVFs) were prepared from biopsies obtained during neck surgery at Aarhus University Hospital as previously described [28]. As the deep neck adipose tissue expresses substantially higher levels of thermogenic markers like UCP1 than the superficial neck adipose tissue [28], we used the superficial subcutaneous neck adipose depot to generate hWAT-SVF and the deep neck adipose depot around the thyroid gland for generation of hBAT-SVF. The adipose tissue biopsies were digested using 0.15 g/ml collagenase (Worthington Biochemical Corporation) in order to separate adipocytes from the SVF. The digestion was under intermittent shaking for 50 min in a buffer containing 2.5 mg/ml bovine serum albumin (BSA) (Sigma-Aldrich). The resulting mixture was filtered through a fine mesh and washed several times. Floating cells were considered mature adipocytes and were collected and snap frozen in liquid nitrogen for later RNA extraction. The SVF was collected from the washing buffers, washed, and pelleted by centrifuging and snap frozen in Dulbecco’s Modified Eagle’s Medium (DMEM) with 5% dimethyl sulfoxide (Sigma-Aldrich) and 50% fetal bovine serum (FBS) (Life Technologies) [29]. The present patient, who had given written consent, was surgically treated for a benign thyroid adenoma and had normal thyroid hormone levels (see also Fig 1). Primary SVF cultures were immortalized with pBabe-puro-TERT as described below. The collection of human biopsies was approved by the Central Denmark Region ethics committee and was performed in accordance with the Declaration of Helsinki.

### Cell culture

TERT-hBA and TERT-hWA cells were cultured in Advanced DMEM/F12 (Life Technologies) supplemented with 10% FBS, L-glutamine (2 mM) (Life Technologies), penicillin (62.5 μg/ml), streptomycin (100 μg/ml) (Sigma-Aldrich), and basic fibroblast growth factor (bFGF) (2.5 ng/ml) (Life Technologies). Two days post-confluent cells (designated day 0) were induced to differentiate in Advanced DMEM/F12 supplemented with 2% FBS, L-glutamine (2 mM), penicillin (62.5 μg/ml), streptomycin (100 μg/ml), insulin (5 μg/ml), dexamethasone (1 μM) (Sigma-Aldrich), 3-isobutyl-1-methylxanthine (IBMX) (0.5 mM) (Sigma-Aldrich), rosiglitazone (1 μM) (Cayman Chemical), human cortisol (1 μM) (Sigma-Aldrich) and T_3_ (1 nM) (Sigma-Aldrich). At day 3, the medium was refreshed with the same medium used at day 0. At day 6 and 9 of differentiation, IBMX, dexamethasone, insulin, rosiglitazone and cortisol were omitted from the medium. At day 12, the adipocytes were considered mature. hMADS [25] and SGBS [22] cells were cultured and differentiated exactly as TERT-hBA and TERT-hWA cells, except that SGBS cells were not treated with bFGF prior to day 0. TERT-hWA, hMADS and SGBS cells used for rosiglitazone-induced browning were differentiated until day 12 as described above, except that T_3_ was omitted from day 6. At day 12, mature TERT-hWA were treated with vehicle or rosiglitazone (1 μM) and harvested at day 15. The cells were kept at 37°C in a humidified atmosphere with 5% CO_2_.

### Retrovirus production and transduction

The retroviral vector pBabe-puro-TERT was kindly provided by Dr. Reuven Agami. Virus packaging was performed in Phoenix-Ampho cells (kindly provided by Dr. Karsten Kristiansen) grown in DMEM (Sigma-Aldrich) containing 10% FBS, supplemented with penicillin and streptomycin, except during transfection. Phoenix-Ampho cells were transfected with the retroviral vector at 50–60% confluence using the FuGENE HD Transfection Reagent (Promega). After 24 h, the medium was changed to regular medium containing antibiotics. Forty-eight h after transfection, the virus-containing cell culture medium was harvested and filtered. Primary human SVFs were transduced three times with 12 h intervals with the virus-containing medium diluted 1:1 with DMEM containing 10% FBS, penicillin, streptomycin and Polybrene (4.5 μg/ml) (Sigma-Aldrich).

### Immunofluorescence staining

Pre-adipocytes were seeded on glass coverslips (10 mm diameter coverslip in 12-well plates) and differentiated as described above. At day 12 of differentiation, the cells were fixed in 4% paraformaldehyde (Sigma-Aldrich) for 10 min at room temperature and washed twice with phosphate-buffered saline (PBS) (Life Technologies). Permeabilization was performed with a 0.5% Triton X-100 (Sigma-Aldrich)/PBS solution for 10 min at room temperature. Permeabilization buffer was aspirated and cells were saturated in a 0.5% BSA /0.1% Triton X-100/PBS solution for 30 min at room temperature. Cells were washed once with PBS before incubation with primary antibody (diluted in 0.5% BSA/PBS) overnight at 4°C. The following day, the cells were washed twice with PBS before incubation with secondary antibody for 30 min at room temperature. After washing, the coverslips were mounted with Aqueous Mounting Medium (DAKO).

### Population doubling time

Eighty percent confluent TERT-hBA and TERT-hWA pre-adipocytes were seeded in 6-well plates at low density. The cells were counted 24 h, 48 h and 72 h after re-plating. Population doubling time was calculated using the following formula:

$$Doubling\;time=\frac{duration\;\left(days\right)x\;\text{log}\left(2\right)}{\text{log}\left(final\;concentration\right)-\text{log}\left(initial\;concentration\right)}$$

### Oil red O staining

Dishes were washed twice with PBS and cells were fixed in 3.7% formaldehyde for 1 h. After aspiration of the formaldehyde, the cells were stained with Oil red O for 1 h. Oil red O was prepared by dissolving 0.5 g Oil red O (Sigma-Aldrich) in 100 ml 2-propanol (Sigma-Aldrich) and diluting it 3:2 with water, followed by filtration. After staining, dishes were washed carefully with PBS and covered with water until photographed.

### Oxygen consumption and extracellular acidification measurements

Real-time oxygen consumption and extracellular acidification rates (OCR and ECAR, respectively) were assessed using a Seahorse XF96 Extracellular Flux Analyzer (Agilent Technologies). Mature TERT-hBA and TERT-hWA adipocytes at day 9 were replated into 96-well XF Cell Culture Microplates (Agilent Technologies) at a density of 20,000 cells per well, essentially as described [30]. Cells were kept in growth medium for two days. At day 11, the cell culture medium was changed 1 h before the first measurement to DMEM (Agilent Technologies) (without serum) supplemented with 5 mM glucose (Sigma-Aldrich) and adjusted to pH 7.4. OCR and ECAR was measured under basal conditions and following injection of isoproterenol (ISO) (10 μM) (Sigma-Aldrich), forskolin (FSK) (10 μM) (Sigma-Aldrich), oligomycin (5 μM) (Agilent Technologies), FCCP (1 μM) (Agilent Technologies) and rotenone/antimycin A (1 μM) (Agilent Technologies). If stated, the cell culture medium contained 2% BSA.

### Gene expression

Isolation of total RNA, reverse transcription and RT-qPCR was done as described [31], except that the SensiFAST SYBR Lo-ROX Kit (Bioline) was used. Primers used for RT-qPCR are listed in S1 Table.

### Immunoblotting

Preparation of whole-cell extracts and immunoblotting were done essentially as described [32]. Briefly, protein lysates were separated on NuPAGE 4–12% Bis-Tris gradient gels (Life Technologies) using NuPAGE MOPS SDS Running Buffer (Life Technologies) and transferred by semi-dry blotting onto polyvinylidene diflouride membrane (GE Healthcare). Equal loading and transfer were confirmed by Amido Black staining (Sigma Aldrich). All washing and incubation steps were carried out with Tris-buffered saline containing 0.1% Tween 20 and 5% non-fat dry milk or BSA. Primary antibodies used were: AKT (#9272), phospho-CREB (Ser133) (#9198), phospho-GSK3α (Ser21) (#9316), phospho-GSK3β (Ser9) (#5558), phospho-p38 MAPK (Thr180/Tyr182) (#9211), phospho-MKK3/6 (Ser189/Ser207) (#12280), phospho-HSL (Ser660) (#4126), phospho-HSL (Ser563) (#4139), phospho-(Ser/Thr) PKA substrate (#9621) (all from Cell Signaling Technology), TFIIB (#sc-225) (Santa Cruz Biotechnology), CYC1 (#sc-7159) (Santa Cruz Biotechnology), FABP4 (#10004944) (Cayman Chemical) and UCP1 (#10983) (Abcam). Secondary antibodies were horseradish peroxidase-conjugated anti-rabbit or anti-mouse (DAKO). EZ-ECL Enhanced Chemiluminescence Detection Kit for HRP (Biological Industries) was used for detection.

### Statistics

Statistical analysis for assessing differences in mRNA levels was performed using GraphPad Prism software. Data are presented as mean, and error bars represent + standard error of the mean (SEM). Statistics was performed on log-transformed data and paired two-tailed Student’s t-tests were used for comparison between the TERT-hBA and TERT-hWA cell models at different passages. Unpaired two-tailed Student’s t-tests were used for comparisons within the same cell model. A p-value of 0.05 was considered statistically significant.

## Results

### Subject and biopsy characterization

To generate human BAT and WAT pre-adipocyte cell models from the same patient, we obtained biopsies from a 48-year old female patient diagnosed with benign thyroid adenoma (Fig 1A). The WAT biopsy was taken from superficial neck fat just above the clavicula and the BAT biopsy was taken from deep neck fat around the thyroid gland [28]. Biopsies were separated into a mature adipocyte fraction (designated hWAT and hBAT, respectively, in Fig 1B and 1C) and an SVF. The SVF was plated and immortalized with retrovirus encoding human TERT to establish cultures called TERT-hWA and TERT-hBA. We chose this immortalization strategy, as it is not possible to immortalize mature adipocytes directly from human biopsy material. To investigate the cellular identity of our biopsy material, we measured thermogenesis-related genes distinguishing thermogenic adipocytes from white adipocytes in the mature adipocyte fractions. *UCP1* mRNA levels were detectable only in hBAT and levels of PPARγ co-activator 1α (*PPARGC1A)* and early B-cell factor 2 (*EBF2*) were markedly higher in hBAT compared to hWAT (Fig 1B). The nucleus-encoded mitochondrial marker genes carnitine palmitoyltransferase 1b, muscle (*CPT1B*) and citrate synthase (*CS*) as well as the mitochondrial DNA (mtDNA)-encoded cytochrome c oxidase II (*COXII*) were expressed at 2-4-fold higher levels in hBAT compared to hWAT (Fig 1C).

In summary, these findings strongly suggest that the biopsy material from which TERT-hWA and TERT-hBA cells is derived is indeed from WAT and BAT, respectively.

### Immortalized human neck pre-adipocytes differentiate into mature adipocytes

**Fig 2:**
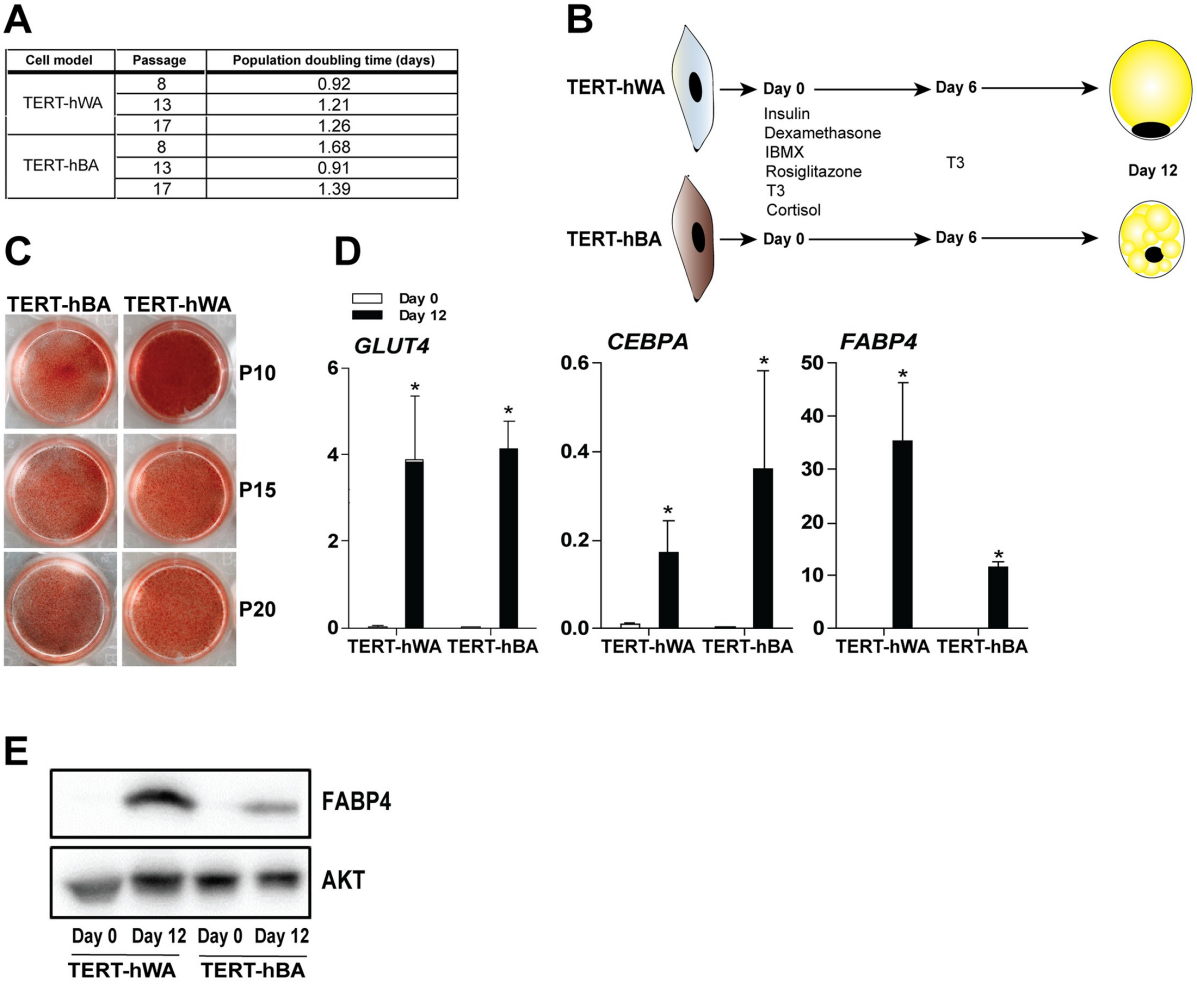
Immortalized human neck pre-adipocytes differentiate into mature adipocytes. **(A)** Population doubling time for TERT-hWA and TERT-hBA pre-adipocytes in passage 8, 13 and 17. **(B)** The general differentiation protocol for TERT-hBA and TERT-hWA. **(C)** Representative overview photos of Oil red O-stained TERT-hBA and TERT-hWA adipocytes at day 12 at passage 10 (P10), 15 (P15) and 20 (P20). **(D)** Relative mRNA levels of the differentiation markers *GLUT4*, *CEBPA* and *FABP4* in pre-adipocytes (day 0) and mature adipocytes (day 12) at P10, P15 and P20. Expression levels were normalized to *TBP* levels. RT-qPCR data are represented as mean of means +SEM from 4 independent experiments (two experiments in P10 and one experiment in P15 and P20). Statistical significance was determined by paired two-tailed Student’s t-test. *, p < 0.05 versus pre-adipocytes (day 0). **(E)** Protein levels of FABP4 in TERT-WA and TERT-hBA pre-adipocytes (day 0) and adipocytes (day 12) at P9. AKT was used as a loading control.

To evaluate basal pre-adipocyte characteristics, we passaged TERT-hBA and TERT-hWA pre-adipocytes up to passage 20 to determine their *in vitro* proliferative capacity. The cells maintained a fibroblast-like morphology with a doubling time of approximately 1–2 days at all passages studied (Fig 2A). We further determined the adipogenic capacity of TERT-hBA and TERT-hWA pre-adipocytes in passage 10, 15 and 20. The general differentiation protocol for TERT-hBA and TERT-hWA is depicted in Fig 2B. The differentiation of TERT-hBA and TERT-hWA cells revealed that a high percentage of pre-adipocytes became lipid-laden, as assessed by Oil red O staining (Fig 2C), indicating the presence of a relatively pure population of adipogenic precursor cells. Both TERT-hWA and TERT-hBA adipocytes exhibited a multilocular morphology (S1 Fig). Differentiation was associated with high expression of the adipogenic differentiation marker genes fatty acid-binding protein 4 (*FABP4*), CCAAT/enhancer-binding protein α (*CEBPA)* and glucose transporter 4 (*GLUT4*) (Fig 2D). FABP4 protein levels were also markedly increased from day 0 to day 12 in both TERT-hBA and TERT-hWA (Fig 2E).

### TERT-hBA adipocytes display features of thermogenic adipocytes

**Fig 3:**
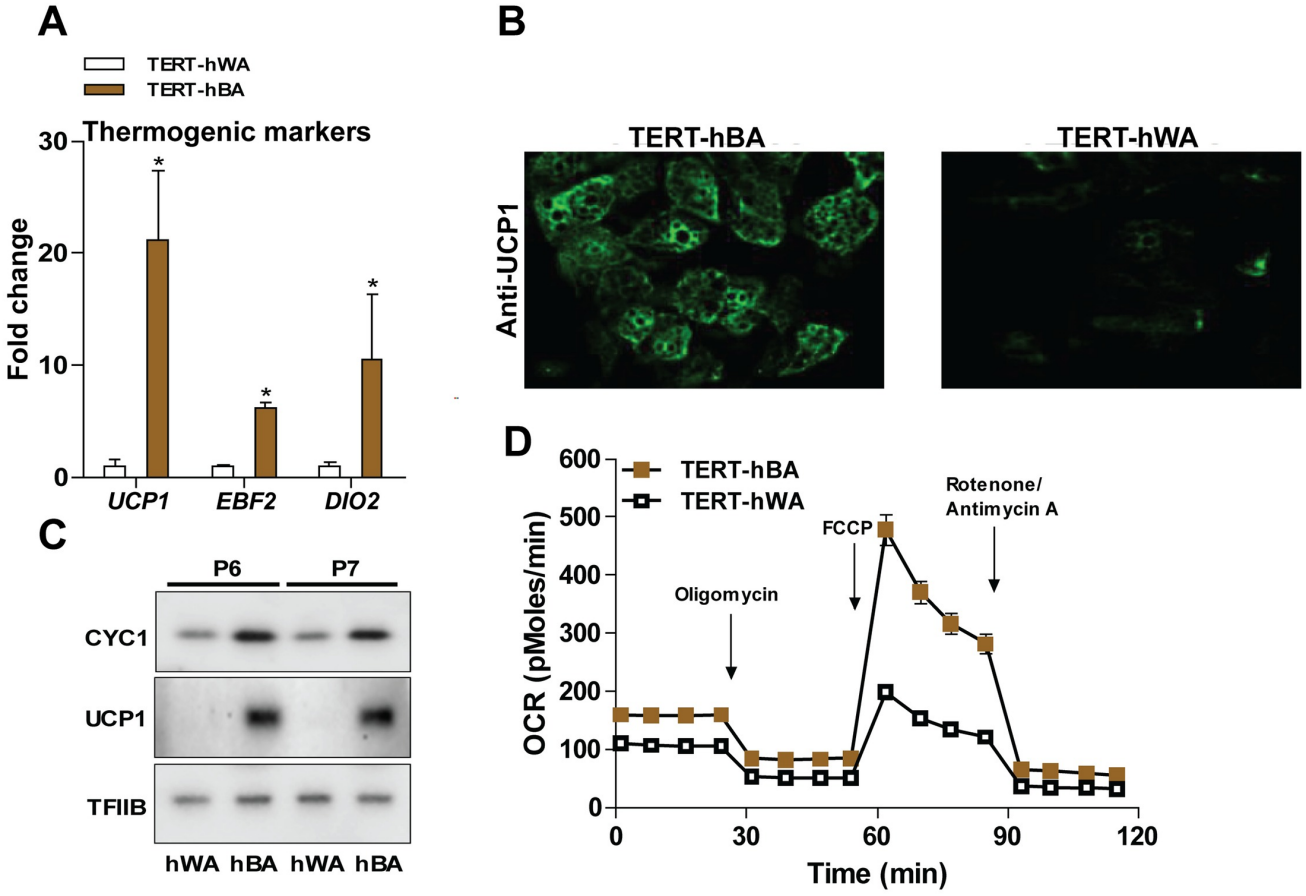
TERT-hBA display features of thermogenic adipocytes. **(A)** Relative mRNA levels of the thermogenic marker genes *UCP1*, *EBF2* and *DIO2* in mature TERT-hBA and TERT-hWA adipocytes (day 12) at passage 10, 15 and 20. Expression levels were normalized to *TBP* levels. The normalized expression in TERT-hWA adipocytes was set to 1. RT-qPCR data are presented as mean of means +SEM from 5 independent experiments (two experiments in passage 10 and passage 15 and one experiment in passage 20). Statistical significance was determined by paired two-tailed Student’s t-test. *, p < 0.05 versus TERT-hWA. **(B)** Immunocytochemistry for UCP1 in mature TERT-hBA and TERT-hWA adipocytes (day 12, passage 5). **(C)** Protein levels of CYC1 and UCP1 in mature TERT-hBA and TERT-hWA adipocytes [day 12, passage 6 (P6) and 7 (P7)]. TFIIB was used as a loading control. **(D)** Time course of oxygen consumption rates (OCR) of mature TERT-hBA and TERT-hWA adipocytes (day 11, passage 7), after sequential injection of oligomycin (5 μM), FCCP (1 μM), rotenone/antimycin A (1 μM of each). Cell culture media contained 2% BSA. Seahorse data are presented as mean +/- SEM of one representative experiment done with 9–12 wells per condition.

Consistent with thermogenic markers being enriched in the adipose fraction from the deep neck biopsy, the thermogenic markers *UCP1*, *EBF2* and *DIO2* were enriched in mature TERT-hBA adipocytes compared with mature TERT-hWA adipocytes in passage 10, 15 and 20 (Fig 3A). These genes are indicative of both brown and brown-like adipocyte identity, and therefore, we measured the proposed brown adipocyte-selective marker genes zinc finger transcription factor-1 (ZIC1) and LIM homeobox 8 (LHX8) [33] and the proposed brown-like-selective markers TNF receptor superfamily member 9 (CD139) and transmembrane 26 (TMEM26) [7]. Co-expression of brown and brown-like adipocyte markers was identified in the adipocyte fraction of the deep neck biopsy (hBAT) (S2A Fig), while TERT-hBA adipocytes exhibited enriched expression of only *ZIC1* and *LHX8* expression in comparison with TERT-hWA adipocytes (S2B Fig). In accordance with the mRNA levels of *UCP1*, we observed higher protein levels of UCP1 in TERT-hBA adipocytes compared to TERT-hWA adipocytes using both immunocytochemistry and immunoblotting (Fig 3B and 3C). Similarly, cytochrome c-1 (CYC1) protein was present at higher levels in TERT-hBA adipocytes (Fig 3C). To further test whether TERT-hBA adipocytes, relative to TERT-hWA adipocytes, possess features of thermogenic adipocytes, we assessed mitochondrial function of the cell models using the Seahorse XF Flux Analyzer with sequential addition of oligomycin, FCCP and rotenone/antimycin A. Substantially larger maximal respiration and spare respiratory capacity were observed in TERT-hBA adipocytes, reflecting a higher mitochondrial capacity of TERT-hBA adipocytes (Fig 3D).

Taken together, these findings suggest that TERT-hBA adipocytes display characteristics of thermogenic adipocytes at both molecular and biochemical levels.

### Response to β-adrenergic stimulation

**Fig 4:**
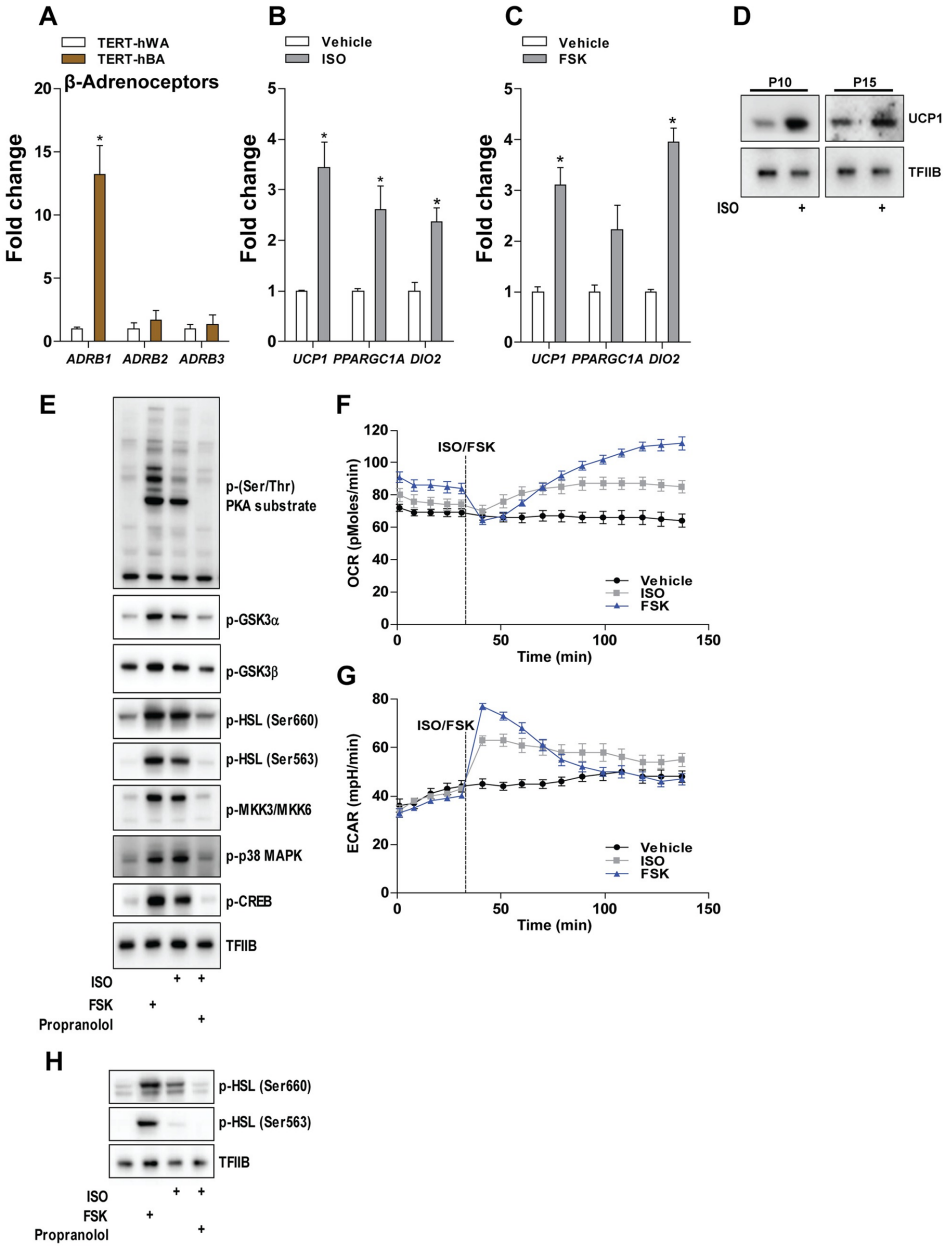
Response to β-adrenergic stimulation. **(A)** Relative mRNA levels of the β-adrenoceptors *ADRB1-3* in mature TERT-hBA and TERT-hWA adipocytes at passage 10, 15 and 20. Expression levels were normalized to *TBP* levels. The normalized expression in TERT-hWA adipocytes was set to 1. RT-qPCR data are presented as mean of means +SEM from 5 independent experiments (two experiments in passage 10 and passage 15 and one experiment in passage 20). Statistical significance was determined by paired two-tailed Student’s t-test. *, p < 0.05 versus TERT-hWA. **(B)** Relative mRNA levels of thermogenic genes in mature TERT-hBA adipocytes (day 12, passage 12) stimulated with 0.1 μM ISO for 6 h. **(C)** Relative mRNA levels of thermogenic genes in mature TERT-hBA adipocytes (day 12, passage 13) stimulated with 10 μM FSK for 6 h. In (B) and (C), expression levels were normalized to *TBP* levels. The normalized expression in vehicle-treated cells was set to 1. Data are presented as mean +SEM of one representative experiment done in technical triplicate. Statistical significance was determined by unpaired two-tailed Student’s t-test. *, p < 0.05 versus vehicle-treated cells. **(D)** UCP1 protein levels in mature TERT-hBA [day 12, passage 10 (P10) and 15 (P15)] stimulated with 0.1 μM ISO for 24 h. TFIIB was used as a loading control. **(E)** Western blot analysis for phosphorylated adipocyte mediators in mature TERT-hBA adipocytes (day 12, P9) pretreated with 10 μM propranolol or vehicle for 1 h before being stimulated with 10 μM FSK or 0.1 μM ISO for an additional 1 h. **(F-G)** Representative time course of oxygen consumption and extracellular acidification rates (OCR and ECAR, respectively) in mature TERT-hBA adipocytes (day 12, passage 9) before and after injection of 10 μM ISO or 10 μM FSK. Data are presented as mean +/- SEM of one representative experiment with 9–12 wells per condition. (H) Western blot analysis for phosphorylated HSL in mature TERT-hWA adipocytes (day 12, passage 9) pretreated with 10 μM propranolol or vehicle for 1 h before being stimulated with 10 μM FSK or 0.1 μM ISO for an additional 1 h.

A key feature of thermogenic adipocytes is being receptive and responsive to β-adrenergic stimuli. To evaluate the receptiveness potential of TERT-hBA adipocytes to β-adrenergic stimuli, we measured the expression of the three β-adrenoceptors. Interestingly, *ADRB1* was expressed at significantly higher levels in TERT-hBA adipocytes compared to TERT-hWA adipocytes, while expression of *ADRB2* and *ADRB3* was similar in TERT-hBA and TERT-hWA cells (Fig 4A). Having established the presence of β-adrenoceptor mRNA in TERT-hBA adipocytes, we stimulated the cells for 6 h with the pan-β-adrenoceptor agonist ISO or FSK, an activator of the adenylate cyclase. FSK was used as a positive control, as it increases intracellular cAMP independent of β-adrenoceptors. TERT-hBA adipocytes responded to ISO and FSK by increasing the expression of *UCP1*, *PPARGC1A* and *DIO2* (Fig 4B and 4C). Notably, UCP1 protein levels were also increased in TERT-hBA adipocytes after treatment with ISO for 24 h (Fig 4D). To further examine the responsiveness of TERT-hBA adipocytes to β-adrenergic stimulation and cAMP, we treated the cells for 1 h with ISO or FSK. As shown in Fig 4E, stimulation with ISO or FSK elicited robust phosphorylation of well-established thermogenic signaling components, such as MKK3/MKK6, p38 MAPK and cAMP responsive element-binding protein (CREB), but also glycogen synthase kinase 3α (GSK3α) and GSK3β, the latter two of which were recently reported to become phosphorylated in response to ISO [34]. As expected, PKA activity was increased by treatment of ISO or FSK, as observed by phosphorylation of hormone sensitive lipase (HSL) at two different amino acid residues and phosphorylation of other PKA substrates. Moreover, pretreatment with the β-adrenoceptor antagonist propranolol abrogated the effect of ISO on the phosphorylation of all signaling components (Fig 4E). In agreement with the receptiveness and responsiveness to β-adrenergic stimulation, TERT-hBA adipocytes increased their oxygen consumption rate after addition of ISO or FSK (Fig 4F). In addition, the extracellular acidification rate transiently increased after addition of ISO and FSK, reflecting an augmented extrusion of protons into the medium and suggestive of an enhanced glycolytic flux (Fig 4G).

Taken together, TERT-hBA adipocytes are receptive and responsive to β-adrenergic stimulation, as demonstrated by upregulation of thermogenic genes and oxygen consumption rate after stimulation with ISO.

A key feature of white adipocytes is the ability to initiate lipolysis in response to β-adrenergic stimulation. Acute stimulation of TERT-hWA adipocytes with ISO or FSK induced the phosphorylation of HSL, the effect of ISO being prevented by pretreatment with propranolol (Fig 4H).

### Conversion of mature TERT-hWA adipocytes into brown-like adipocytes upon exposure to rosiglitazone

**Fig 5:**
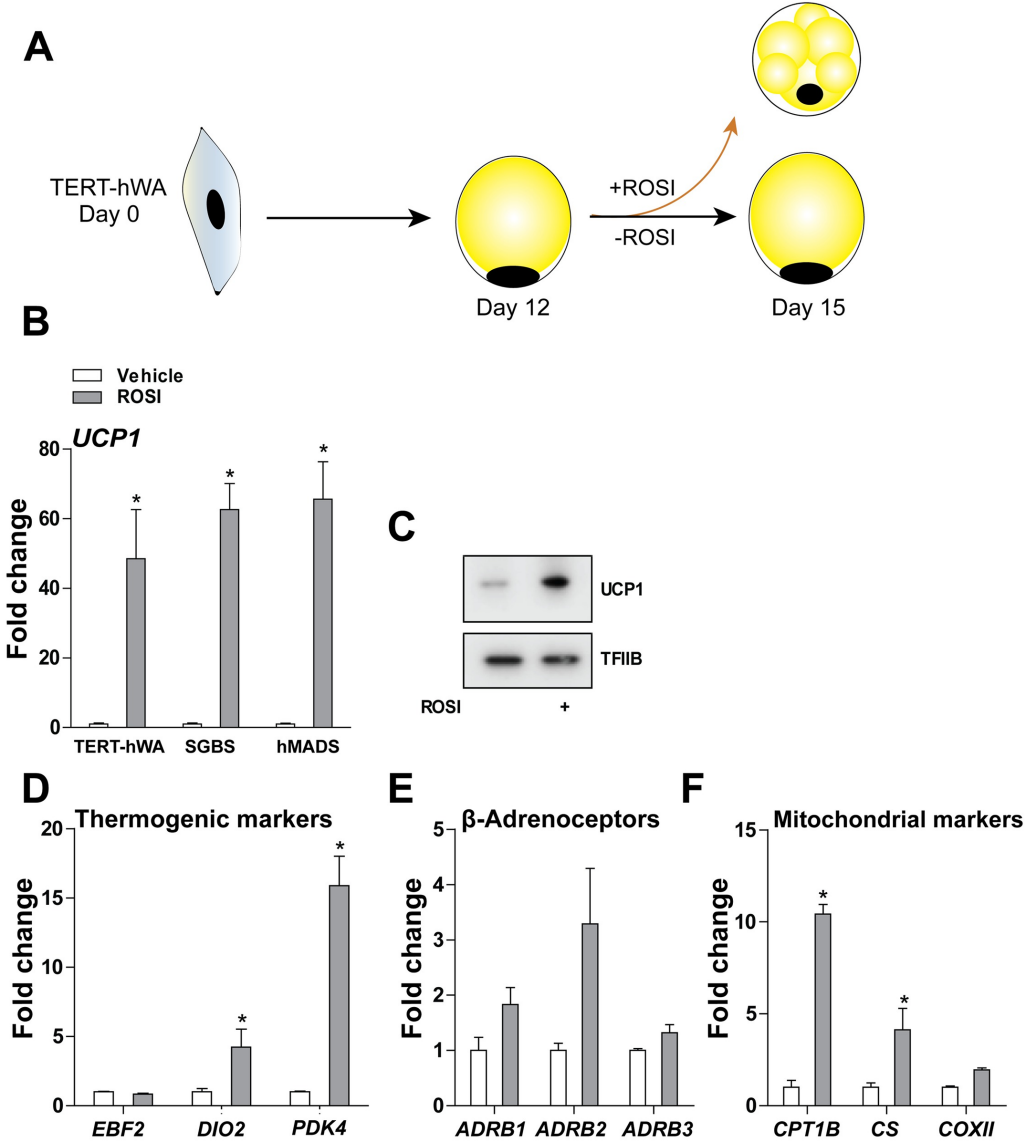
Conversion of mature TERT-hWA adipocytes into brown-like adipocytes upon exposure to rosiglitazone. **(A)** The rosiglitazone-induced browning protocol. **(B)** Relative mRNA levels of *UCP1* in mature TERT-hWA adipocytes (day 15, passage 7–14), SGBS and hMADS adipocytes exposed to vehicle or 1 μM rosiglitazone from day 12 to 15. **(C)** Protein levels of UCP1 in TERT-hWA adipocytes (day 15, passage 13) exposed to vehicle or 1 μM rosiglitazone from day 12 to 15. TFIIB was used as a loading control. **(D)** Relative mRNA levels of the thermogenesis-related genes *EBF2*, *DIO2* and *PDK4* in mature TERT-hWA adipocytes (day 15, passage 12) exposed to vehicle or 1 μM rosiglitazone from day 12 to 15. **(E)** Relative mRNA levels of the β-adrenoceptors *ADRB1-3* in mature TERT-hWA adipocytes (day 15, passage 12) exposed to vehicle or 1 μM rosiglitazone from day 12 to 15. **(F)** Relative mRNA levels of the mitochondrial markers *CPT1B*, *CS* and *COXII* in mature TERT-hWA adipocytes (day 15, passage 12) exposed to vehicle or 1 μM rosiglitazone from day 12 to 15. In (B) and (D-F), expression levels were normalized to *TBP* levels. The normalized expression in vehicle-treated cells was set to 1. Data are presented as mean +SEM of one representative experiment done in technical triplicate. Statistical significance was determined by unpaired two-tailed Student’s t-test. *, p < 0.05 versus vehicle-treated cells.

To examine the potential of TERT-hWA cells to induce a brown-like adipocyte program, we exposed mature TERT-hWA adipocytes to either β-adrenergic stimulation (ISO) or rosiglitazone from day 12 to 15. In contrast to rosiglitazone, this long-term treatment with ISO (refreshed every 24 h) had no effect on the expression of thermogenic marker genes (data not shown). To expand on this observation, we compared TERT-hWA to two other human white adipocyte cell models (SGBS and hMADS) in terms of rosiglitazone-induced browning, as depictured in Fig 5A. The three cell models were cultured under identical conditions from day 0 to 15. As previously reported in white adipocyte cell models, including hMADS, rosiglitazone induced the expression of *UCP1* (Fig 5B) [35–38]. TERT-hWA increased UCP1 mRNA to the same extent as SGBS and hMADS (Fig 5A). UCP1 protein levels were also increased after rosiglitazone treatment of TERT-hWA (Fig 5C). Rosiglitazone also increased the expression of *DIO2* and pyruvate dehydrogenase kinase 4 (*PDK4*), but not *EBF2* (Fig 5D), while the expression of β-adrenoceptors only tended to increase in response to rosiglitazone (Fig 5E). *CPT1B* and *CS* mRNA levels were significantly increased by rosiglitazone-induced browning, indicative of augmented mitochondrial function (Fig 5F). Taken together, these results demonstrate that mature TERT-hWA adipocytes can convert into brown-like adipocytes upon exposure to rosiglitazone.

## Discussion

Mice represent a powerful experimental tool to decipher the complexity of adipocyte biology. Indeed, much of our current conceptualization of the regulation of adipocyte function in humans is influenced by insight obtained from mouse models. A potential concern is the extent to which mouse models faithfully mimic the human situation. Differential responses of human and mouse adipocytes to glucocorticoids [17], retinoic acid [18], adrenocorticotropic hormone [19] and tumor necrosis factor [20] suggest that extrapolation from one species to another should be carried out with caution. The field of BAT research received renewed focus in 2009 with the finding that adult humans possess active BAT. Since then, mouse models have revealed a wealth of information on the molecular control of formation, function and therapeutic potential of BAT [39].

In this study, we report on the generation of a set of polyclonal, immortalized cell models from WAT and BAT from the cervical region from the same donor. Due to the polyclonal nature of the cell models, it is important to keep in mind that the cultures likely contain cell types other than pre-adipocytes. The cell models maintained a fibroblast-like morphology during propagation and were passaged in culture for more than 20 passages. TERT-hBA and TERT-hWA pre-adipocytes robustly differentiated into mature adipocytes that recapitulated key features of thermogenic and white adipocytes, respectively (Figs 2–4, S1 Fig). The specific isolation of human classical brown versus brown-like adipocytes from biopsy material can be challenging due to poor accessibility and anatomical diffuseness of BAT. The biopsy material to generate TERT-hBA cells was obtained from the deep neck adipose tissue around the thyroid gland, an area that has been suggested to exhibit a heterogeneous population of classical brown and brown-like adipocytes [5, 6]. We attempted to determine if the TERT-hBA cells resembled the identity of brown or brown-like adipocytes. To achieve that, we measured the expression of previously proposed adipocyte type-selective identity marker genes in the adipocyte fraction from the biopsy as well as in the derived cell models analyzed in several passages. The adipocyte fraction from the BAT biopsy displayed markedly higher expression of known thermogenesis-related genes (*UCP1*, *PPARGC1A* and *EBF2*) and genes encoding mitochondrial proteins (*CTP1B*, *CS* and *COXII*) (Fig 1), in comparison to the adipocyte fraction from the WAT biopsy obtained from the superficial cervical adipose tissue. This is consistent with the biopsies representing thermogenic and white adipose tissue, respectively. In the BAT biopsy material, we identified the coexistence of classical brown and brown-like adipocyte identity marker genes, consistent with previous reports [5, 6]. Interestingly, in the immortalized cell models, the brown adipocyte-enriched markers *ZIC1* and *LHX8* were expressed at higher levels in differentiated TERT-hBA adipocytes than in TERT-hWA adipocytes (S2 Fig), while two proposed brown-like adipocyte-selective markers, *TMEM26* and *CD137*, were unable to discriminate between TERT-hBA and TERT-hWA adipocytes (S2 Fig). Thus, expression of the four adipocyte type-selective marker genes suggested that whereas the original biopsy was composed of a mixture of brown and brown-like adipocytes, the derived TERT-hBA cells more closely resembled brown adipocytes. However, it is important to note that the adipocyte type identity marker genes are relative rather than absolute, and they are unlikely to provide unequivocal evidence for a brown or brown-like adipocyte origin. To support a distinction based on gene expression, metabolic differences could be investigated. It has been reported that the mouse brown-like adipocyte mitochondrion has a lower capacity to utilize glycerol-3-phosphate relative to the brown adipocyte mitochondrion [40], and it was recently elucidated that mouse brown-like adipocytes, but not brown adipocytes, use creatine to dissipate energy when ADP is limiting [41]. It remains to be shown if TERT-hBA adipocytes metabolically resemble brown or brown-like adipocytes.

Mature TERT-hBA adipocytes displayed receptiveness and responsiveness to β-adrenergic stimulation, as demonstrated by the presence of the *ADRB1-3* mRNAs, as well as ISO-induced activation of intracellular signaling supporting thermogenesis, induction of thermogenic gene expression and increased oxygen consumption, and mature TERT-hWA adipocytes responded to ISO stimulation by increasing activating phosphorylations of HSL (Fig 4). Moreover, TERT-hWA adipocytes, like hMADS and SGBS adipocytes, underwent efficient rosiglitazone-induced browning, demonstrating the capability to activate a brown-like adipocyte gene program in these cells (Fig 5).

Human primary pre-adipocyte cultures are excellent models for studying cell-autonomous adipocyte biology. Despite this, a major bottleneck for human BAT research is the limited accessibility of biopsy material and the mortal nature of primary pre-adipocyte cultures. In an attempt to overcome this challenge, we expanded the proliferative lifespan of primary cells by overexpressing the catalytic subunit of human TERT. Using this strategy, we show that it is feasible to obtain enough material from a single patient to establish immortalized, polyclonal cell models of human cervical BAT and WAT, thereby eliminating donor-to-donor variation. Contrary, the fact that the TERT-hBA and TERT-hWA cell models derive from a single donor, leaves open the possibility that their differentiation and/or response to stimuli are not ‘representative‘ of the adipocyte type they are expected to reflect. The polyclonal nature of the cell models will allow further investigations into human brown adipocyte physiology, but at the same time permit analysis of the heterogeneous nature of human BAT at single-cell resolution by subcloning or novel single-cell sorting technologies.

### Conclusion

In this study, we report the generation of polyclonal, patient-matched human brown and white adipocyte cell models to further explore the regulation and therapeutic potential of BAT and browning of WAT in humans. The generated cell models, TERT-hBA and TERT-hWA, exhibited high proliferative and adipogenic capacity until at least passage 20. Furthermore, they recapitulated the gene expression profile of brown and white adipocytes, respectively. We confirmed that TERT-hBA adipocytes were receptive and responsive to β-adrenergic stimuli with upregulation of thermogenic genes, phosphorylation of signaling mediators and increased respiration.

## Supporting information

S1 Fig Micrographs of TERT-hWA and TERT-hBA pre-adipocytes and adipocytes. Representative micrographs of Oil red O-stained TERT-hBA and TERT-hWA cells at day 0 and 12 at passage 7–9.
(TIF)

S2 Fig Expression of brown and brown-like adipocyte type-selective marker genes. **(A)** Relative mRNA levels of the proposed brown and brown-like adipocyte-selective genes *ZIC1*, *LHX8*, *TMEM26* and *CD137* in hBAT and hWAT. Expression levels were normalized to *TBP* levels. The normalized expression in hWAT was set to 1, except for *TMEM26* in which hBAT was set to 1. Data represent the mean of a technical duplicate without error bars, since only one patient was included. Statistical analyses were not applied. **(B)** Relative mRNA levels of proposed brown and brown-like adipocyte-selective genes in mature TERT-hBA and TERT-hWA adipocytes (day 12) at passage 10, 15 and 20. Expression levels were normalized to *TBP* levels. The normalized expression in vehicle-treated cells was set to 1. Data are presented as mean of means +SEM from 5 independent experiments (two experiments in passage 10 and passage 15 and one experiment in passage 20). Statistical significance was determined by paired two-tailed Student’s t-test. *, p < 0.05 versus TERT-hWA.
(TIF)

S1 Table Primers used for RT-qPCR. (PDF)
