# Supplementary figures and images for: Characterization of immortalized human brown and white pre-adipocyte cell models from a single donor
Source: PLoS One. 2017 Sep 28;12(9):e0185624. doi: 10.1371/journal.pone.0185624 (PMC5619805; doi:10.1371/journal.pone.0185624)

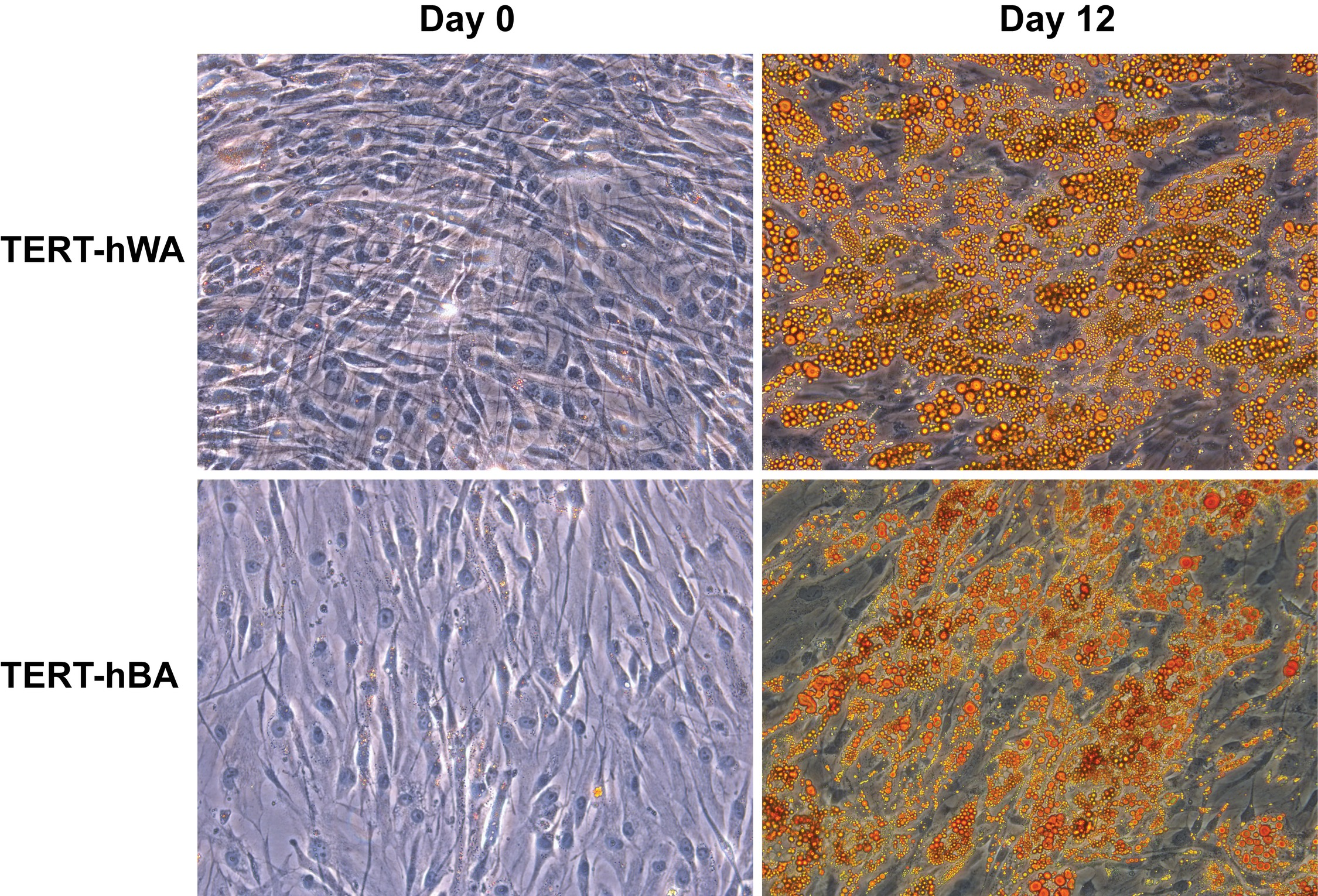

Supplement: S1 Fig — Representative micrographs of Oil red O-stained TERT-hBA and TERT-hWA cells at day 0 and 12 at passage 7–9. (TIF) [file pone.0185624.s001.tif]

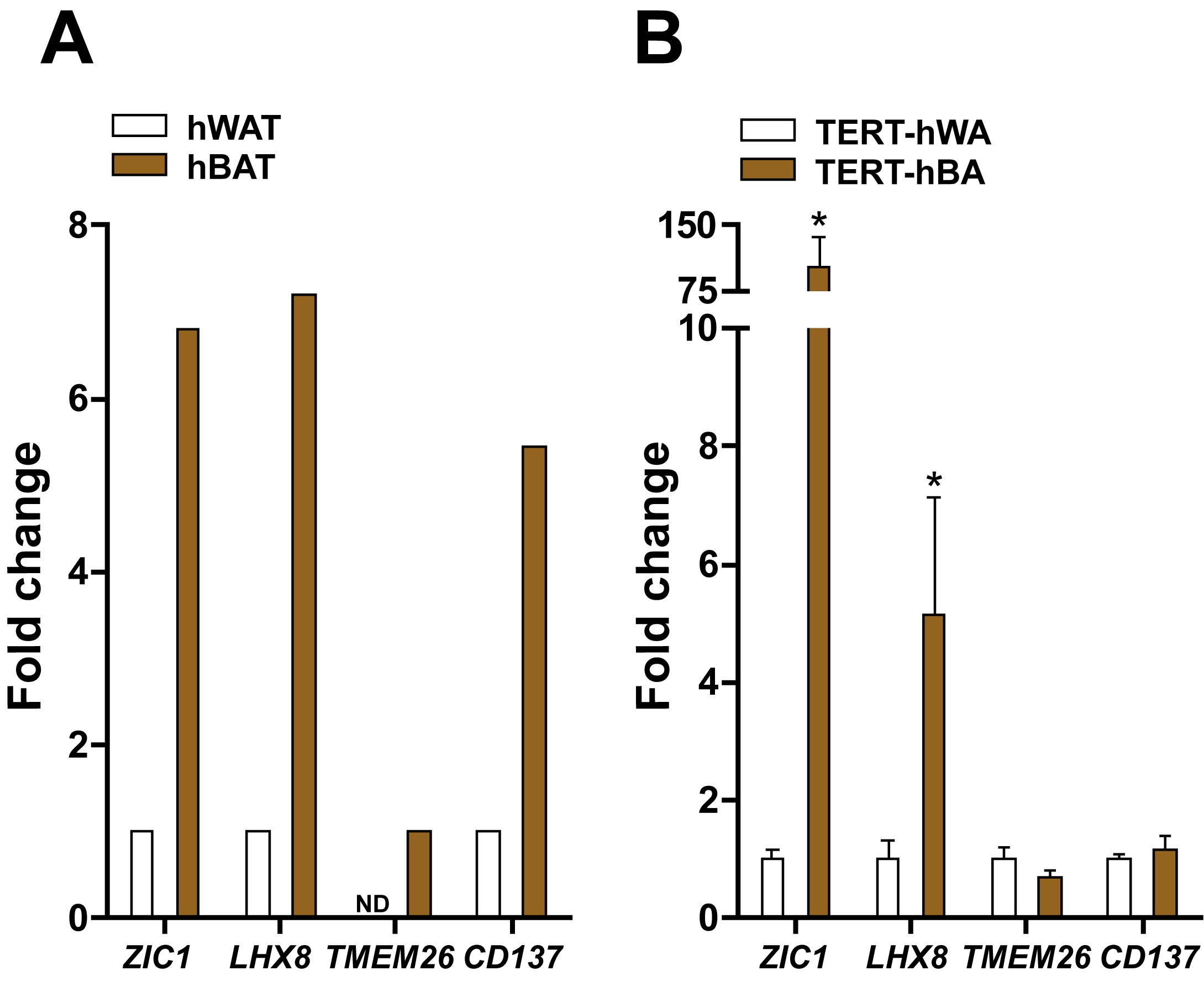

Supplement: S2 Fig — (A) Relative mRNA levels of the proposed brown and brown-like adipocyte-selective genes ZIC1, LHX8, TMEM26 and CD137 in hBAT and hWAT. Expression levels were normalized to TBP levels. The normalized expression in hWAT was set to 1, except for TMEM26 in which hBAT was set to 1. Data represent the mean of a technical duplicate without error bars, since only one patient was included. Statistical analyses were not applied. (B) Relative mRNA levels of proposed brown and brown-like adipocyte-selective genes in mature TERT-hBA and TERT-hWA adipocytes (day 12) at passage 10, 15 and 20. Expression levels were normalized to TBP levels. The normalized expression in vehicle-treated cells was set to 1. Data are presented as mean of means +SEM from 5 independent experiments (two experiments in passage 10 and passage 15 and one experiment in passage 20). Statistical significance was determined by paired two-tailed Student’s t-test. *, p < 0.05 versus TERT-hWA. (TIF) [file pone.0185624.s002.tif]
