# Supplementary material for: Characterization of immortalized human brown and white pre-adipocyte cell models from a single donor
Source: PLoS One. 2017 Sep 28;12(9):e0185624. doi: 10.1371/journal.pone.0185624 (PMC5619805; doi:10.1371/journal.pone.0185624)
Supplement: S1 Table — (PDF) [file pone.0185624.s003.pdf]

Table S1

Gene	Forward primer	Reverse primer
ADRB1	CAGGCTGAGGGATTTCC	GCCTGGTCCTTCCAACTAAT
ADRB2	CTTCTTGCCCATTCAGATGC	AAGAAGTCACAGCAGGTCTC
ADRB3	AGGTTATGCCAATTCTGCCT	CAAGAAGCCCCGTCGAG
CEBPA	AAGAAGTCGGTGGACAAGAAC	GTCATTGTCACTGGTCAGCTC
CD137	CAGGCAGTGTAAAGGTGTTTT	TGCCACGTTTCTGATCGTTA
COXII	AGTCCTGTATGCCCTTTTCCT	TTCAGACGGTTTCTATTTCCTG
CPT1B	CTCCTTTCCTTGCTGAGGTG	TCTCGCCTGCAATCATGTAG
CS	GGACATATCCCAACAGAGGAA	CTCTTTGCCCACTCTTTTGAG
DIO2	ACCACCCACAAAGAAACAAAG	GGCAGAGATAAGCCTTGAATG
EBF2	CGGTTTCAGGTTGTGTTGTC	GGCTAATGGCTTTGATGCAG
FABP4	AGCACCATAACCTTAGATGGGG	CGTGGAAGTGACGCCTTTCA
GLUT4	AACTGGACGAGCAACTTCATC	AGGACCGCAAATAGAAGGAAG
LHX8	GTGGTTGATGAATTATGGCTGT	ACTCAGAATGGTGGGCTTAT
PPARGC1A	CCACAGAGAACAGAAACAGCA	TGGGGTCAGAGGAAGAGATAA
TBP	CCCGAAACGCCGAATATAA	GAAAATCAGTGCCGTGGTTC
TMEM26	ACCATGAGACCCAGTATTGC	GATGGAGTCCCAATGTCCAA
UCP1	CCAACTGTGCAATGAAAGTGT	CAAGTCGCAAGAAGGAAGGTA
ZIC1	CAAACACATGAAGGTCCACG	ATAAGGAGCTTGTGGTCGG
